# Aesthetic discomfort in hand osteoarthritis: results from the LIège Hand Osteoarthritis Cohort (LIHOC)

**DOI:** 10.1186/s13075-015-0807-y

**Published:** 2015-11-30

**Authors:** Audrey Neuprez, Olivier Bruyère, Emmanuel Maheu, Nadia Dardenne, Nansa Burlet, Pieter D’Hooghe, Stéphan Distèche, Jean-Yves Reginster

**Affiliations:** Department of Public Health, Epidemiology and Health Economics, University of Liège, Liège, Belgium; Department of Rheumatology, AP-HP, St. Antoine Hospital, Paris, France; Department of Orthopaedics and Sports Medicine, Aspetar Hospital, Doha, Qatar; Department of Public Health, Epidemiology and Health Economics, University of Liège, Liège, Belgium

**Keywords:** Hand osteoarthritis, Erosive, Aesthetic discomfort, Quality of life

## Abstract

**Introduction:**

The primary complaint of patients with hand osteoarthritis (OA) is frequently the inelegant appearance of their hands. Only one study has been conducted to assess the magnitude of and identify the determinants of aesthetic discomfort in hand OA.

**Methods:**

The LIège Hand Osteoarthritis Cohort is a prospective cohort of 203 patients diagnosed with hand OA. At baseline, these patients rated their aesthetic discomfort on a 100-mm visual analogue scale (VAS) and used a Likert scale (range 0–7) to quantify the magnitude of their aesthetic damage.

**Results:**

The median value of the aesthetic discomfort VAS was 35.0 [interquartile range (Q1–Q3) 6.0–59.0]. The median damage was rated 3.0 (Q1–Q3 1.0–4.0), corresponding to a moderate level. Both were significantly (*p* < 0.02) associated with the female gender, the duration of hand OA, the radiological severity of OA (Verbruggen–Veys and Kellgren–Lawrence scales) and pain, disability, or stiffness [Australian Canadian Osteoarthritis Hand Index (AUSCAN) and Functional Index for Hand Osteoarthritis ]. After a stepwise analysis, the parameters correlated to the aesthetic discomfort were the presence of erosive joints (*p* = 0.0048), the AUSCAN score (*p* < 0.0001), the number of joints with severe radiological damage (*p* = 0.023), and gender (*p* = 0.0009). For aesthetic damage, the parameters associated were AUSCAN score (*p* < 0.0001), duration of hand OA >10 years (*p* = 0.001), and presence of erosive joints (*p* < 0.0001). Compared with patients with low aesthetic discomfort (VAS ≤33 mm), those with the highest discomfort (VAS ≥66 mm) had more erosive OA (*p* = 0.014), a higher Verbruggen and Veys score (*p* = 0.0039), and a higher AUSCAN score (*p* < 0.001).

**Conclusions:**

Aesthetic discomfort and damage are significant complaints in patients with hand OA. The determinants of the magnitude of these are gender, radiological severity, duration of hand OA, presence of erosive joints, and impact on pain, function, and stiffness as assessed with the AUSCAN.

**Electronic supplementary material:**

The online version of this article (doi:10.1186/s13075-015-0807-y) contains supplementary material, which is available to authorized users.

## Introduction

Osteoarthritis (OA) is a disease representing failed repair of joint damage that, in the preponderance of cases, is triggered by abnormal intraarticular stress [1]. OA is the most common form of arthritis and is a major cause of morbidity, activity limitation, physical disability, excess health care use, and reduced health-related quality of life (HRQoL), especially in people aged 45 and older, in developed countries [2]. The prevalence of hand OA (HOA) varies greatly and has been reported to range from 27 % to 80 % [3, 4]. This may be related to race/ethnicity (e.g., lower prevalence of HOA in Chinese compared with Caucasians [5]), the definition of HOA (i.e., based on hand radiology or on physical examination [6]), the scoring method used [7], the specific joints under study, and the characteristics of the study population [8]. In most studies the prevalence of HOA increases with age, and differentiation by gender shows that women are more frequently affected than men [4]. In the Framingham study, the prevalence of symptomatic HOA, defined as having frequent pain in a joint and radiographic evidence of OA in that joint, was 26.2 % in women and 13.4 % in men aged 71 years and older [4]. Data on the burden of HOA and its impact on HRQoL are limited. A systematic review of the literature published in 2011 suggested that HOA may have almost as great an impact as rheumatoid arthritis (RA) on HRQoL [9]. A major impact of HOA on health utility scores compared with healthy controls was also shown in a Scandinavian cohort, which, however, suggested that physical function was more affected by RA than by HOA [10]. Whereas dissatisfaction with hand appearance is frequently the presenting complaint of patients with HOA, no specific tool exists for its measurement and few studies have addressed aesthetic discomfort [9, 11]. Nevertheless, the OARSI recommendations list it as a domain that should be systematically assessed in HOA studies or trials [12]. So far, only one study has suggested that aesthetic discomfort is a major concern for a significant number of patients with HOA, particularly women, those with a high burden of HOA disease, and those with erosive OA; in addition, aesthetic discomfort is associated with depression, anxiety, and poor HRQoL [11].

The objectives of the present study were to assess the magnitude and the determinants of the aesthetic discomfort in HOA using the baseline data of the LIège Hand Osteoarthritis Cohort (LIHOC), a prospective study of 203 patients diagnosed with HOA who are prospectively followed to better understand the impact of HOA on HRQoL and health resource use.

## Material and methods

### Patients and assessments

Between February 2013 and July 2014, 203 consecutive outpatients attending the Bone and Cartilage Metabolism Unit at the University of Liège, Belgium, who were diagnosed with HOA were included in LIHOC. These patients were attending a tertiary care center specialized in the diagnosis, prevention, and treatment of osteoporosis and OA. Among them, 50 % were consulting for an osteoporosis screening and the other half had been referred for low back pain and/or OA-related symptoms. HOA was the chief complaint in <10 % of the population. All patients met the American College of Rheumatology x-ray/clinical criteria for HOA [13]. All patients gave written informed consent, and the study received prior approval from the Liège University Ethics Committee. Demographic and clinical characteristics of the population were recorded according to a standardized case report form and included age, menopausal status in women, highest education level attained, current smoking and alcohol consumption, duration since the onset of HOA symptoms, family history of HOA, OA diagnosed in other joints, weight, height, and body mass index (BMI).

HOA was assessed by a specialist physician (SD) in terms of number of painful (at rest or at pressure) and tender (swollen) joints (performed for all distal and proximal interphalangeal joints and thumb base) and presence of Heberden’s or Bouchard’s nodes. The intrareader test–retest reproducibility of the x-ray reading was tested, and the intraclass correlation coefficient ranged between 0.72 and 0.99 for both the Kellgren and Lawrence scale (K-L) score [14] and the Verbruggen and Veys score [15]. Patient global assessment of pain was recorded using a 100-mm (0–100) visual analogue scale (VAS).

The patients were asked to rate their aesthetic discomfort related to HOA on a 100-mm VAS (0–100) and also on a Likert scale (0–7) commonly used for the assessment of aesthetic damage in forensic (i.e., medicolegal assessment) medicine (ranging from 0 = no damage to 7 = very important damage) [16].

The three dimensions of pain, stiffness, and function were assessed using the VAS version of the Australian Canadian Osteoarthritis Hand Index (AUSCAN; normalized score range 0–300) [17, 18]. Functional disability was also measured using the Functional Index for Hand Osteoarthritis (FIHOA) (range 0–30) [19, 20]. HRQoL was assessed using the 12-item Short Form Health Survey (SF-12) (range 0–100) [21] and the EuroQol (EQ-5D) (range 0–1) [22]. Psychological status was measured with the Hospital Anxiety and Depression scale (HADS; range 0–21 for both anxiety and depression) [23].

Posteroanterior radiographs of both hands were assessed according to the Verbruggen and Veys scale (range 0–218) and the K-L grading scale (range 0–128). We also assessed the presence of erosive OA as defined by Verbruggen and Veys (i.e., at least one joint at erosive or remodeled stage) and the number of severely affected joints using the K-L grading scheme (i.e., K-L grade 4).

### Statistics

Quantitative variables were expressed as median and interquartile range (Q1–Q3), owing to skewed distributions. Qualitative variables were expressed as number and percentage. In the univariate analysis, association between aesthetic discomfort (VAS) or the magnitude of the aesthetic damage (medicolegal scale), and qualitative parameters was assessed by means of a Student’s *t* test or the Kruskal–Wallis test. Correlations with quantitative parameters were tested by using Pearson’s or Spearman’s rank correlation. All parameters with a *p* value <0.25 in the univariate analysis were then combined in a multiple regression with stepwise procedure to account for potential cofounders [24, 25].

As recommended by Hodkinson et al. [11], we conducted a similar analysis to compare patients with either high (VAS ≥66 mm) or low (VAS <33 mm) aesthetic concerns rather than in patients who felt neutral about their hand appearance (34 mm ≤ VAS ≤ 65 mm). The choice of these values was motivated by their clinical relevance: around 33 mm on a VAS, for instance, for pain is the cutoff separating patients at a patient-acceptable symptom state in knee or hip OA versus those with pain. A value >66 mm on a VAS reflects a high magnitude of the dimension measured (pain, functional impairment, or global assessment) [11, 26].

Results were considered to be statistically significant at the 5 % critical level (*p* < 0.05). Data analysis was carried out using the SAS version 9.3 for Windows statistical software package (SAS Institute, Cary, NC, USA).

## Results

A total of 203 patients were analyzed. The median age of the population was 69.1 years (Q1–Q3 61.9–75.6). Of the patients included, 90.1 % were women and 46.8 % had a family history of HOA. The median BMI (kg/m^2^) was 25.6 (Q1–Q3 22.9–28.9). HOA was associated with OA involvement of other joints in 87.1 % of the cases [mainly spine (70.8 %) and knee (69 %)]. The reported durations since the onset of HOA symptoms were <1 year, 1–5 years, 6–10 years, and >10 years, in 6.9 %, 40.4 %, 22.2 %, and 30.5 % of the subjects, respectively. The median score for hand pain at rest on a 100-mm VAS was 50.0 (Q1–Q3 29.0–59.0). The median number of painful joints at rest and with pressure were 1.0 (Q1–Q3 0.0–4.0) and 5.0 (Q1–Q3 2.0–10.0), respectively. The median number of swollen (tender) joints and joints with bony deformations were 2.0 (Q1–Q3 1.0–4.0) and 10.0 (Q1–Q3 6.0–15.0), respectively. Eighty-seven (42.9 %) subjects presented with erosive HOA. The impact of HOA on HRQoL (EQ-5D and SF-12), psychological status (HADS), and functional disability (AUSCAN and FIHOA), as well as radiological severity (Verbruggen–Veys and K-L scores) are summarized in Additional file 1: Table S1.

The median value of the aesthetic discomfort on the 100-mm VAS was 35.0 (Q1–Q3 6.0–59.0). On the Likert medicolegal scale, the median aesthetic damage was rated as 3.0 (Q1–Q3 1.0–4.0), corresponding to a moderate level of damage.

Aesthetic discomfort scores (VAS) presented a trimodal distribution, with 100 patients (49.3 %) grading their discomfort ≤33 mm [low aesthetic discomfort (LAD)], 61 patients (30.0 %) between 34 mm and 65 mm [intermediate aesthetic discomfort (IAD)], and 42 patients (20.7 %) ≥66 mm [high aesthetic discomfort (HAD)].

In the univariate analysis of the entire population, aesthetic discomfort (VAS) and the magnitude of the aesthetic damage (medicolegal scale) were significantly (*p* < 0.02) associated with patient age (medicolegal scale only), patient gender, duration since the onset of HOA symptoms, Verbruggen–Veys and K-L radiological scores, AUSCAN and FIHOA scores, patient global assessment of pain, presence of erosive HOA, number of joints presenting with severe lesions or bony deformations, and number of joints spontaneously reported as painful (medicolegal scale only) or painful with pressure. However, when a stepwise analytical procedure was applied, the only parameters that remained significantly correlated to aesthetic discomfort (VAS) were the number of joints with severe HOA (based on K-L score; *p* = 0.023), the total normalized AUSCAN score (*p* < 0.0001), female gender (*p* = 0.0009), and the presence of erosive HOA (Verbruggen and Veys scale; *p* = 0.0048). For the assessment of aesthetic damage, the parameters that remained significant were the total normalized AUSCAN score (*p* < 0.0001), presence of erosive HOA (Verbruggen and Veys) (*p* < 0.0001), and duration of HOA >10 years (*p* = 0.001).

Additional file 2: Table S2 summarizes the characteristics of the patients reporting HAD and LAD and the outcomes of the univariate analysis comparing these two groups. There were no significant differences between patients reporting HAD or LAD for EQ-5D score, SF-12 physical score, SF-12 mental score, HADS anxiety, and HADS depression (data not shown). The parameters that remained significantly different between the two groups after multivariate analysis were the Verbruggen and Veys radiological score (*p* = 0.0039), total normalized AUSCAN score (*p* < 0.001), and presence of erosive OA (*p* = 0.014).

## Discussion

Whereas aesthetic discomfort is often one of the primary complaints of patients with HOA, few studies have investigated the magnitude and the determinants of this important concern [9–12]. Besides the four questions concerning hand appearance contained in the Michigan Hand Outcomes Questionnaire [27] (which could not be used in our study because of the lack of a validated French translation), no instrument has been developed for the specific assessment of aesthetic discomfort associated with HOA [11]. With the aim of gaining a better understanding of the impact of HOA on HRQoL and health resources use, we took the benefit of recruitment of a prospective cohort (LIHOC) to include at baseline patients’ self-reported assessment of their aesthetic discomfort and a measure of patients’ perceptions of the aesthetic damage generated by HOA. The demographics of our population showed a high proportion of slightly overweight women. All epidemiological studies acknowledge a higher prevalence of HOA in women than in men [4, 28, 29]. The overwhelming majority of our sample were women (>90 %), a pattern also found in other studies [11]. In our present study, this demographic characteristic was most likely related to our tertiary care setting specializing in osteoporosis and osteoarthritis. Hence, our study was oriented toward women more than men. No previous studies have been conducted to investigate whether different perceptions of aesthetic discomfort exist between men and women with HOA. As in the Hodkinson et al. study [11], in our study female sex was significantly associated to a higher degree with aesthetic discomfort. It has previously been suggested that, although more symptomatic in women, radiographic HOA is nearly similar in both genders [30]. Furthermore, due to the relatively small number of men included in our study, the conclusions regarding the impact of sex on aesthetic discomfort should be read with caution. The slightly increased BMI of our population is in accordance with most [29, 31, 32] but not all [11] reports of patients with HOA. BMI did not influence the aesthetic consequences of HOA in our population.

The median value of the aesthetic discomfort related to HOA was measured as 35.0 on a 100-mm VAS, reflecting a significant concern in our sample. The median value of 3.0 on the medicolegal scale is usually associated, in forensic medicine, with damage considered moderate [14]. These values compare rather well with those published in the only previous study in which researchers looked at the aesthetic impact of HOA in a population that, as ours, was constituted mostly of women [10] also recruited in a tertiary specialized HOA clinic. This observation confirms that dissatisfaction with the appearance of the hands is a significant concern for patients with HOA and represents a currently unmet medical need.

The significant determinants of aesthetic discomfort induced by changes in the appearance of the fingers were, in our multivariate analysis, the presence of erosive joints; the number of joints graded on the radiological K-L scale as severely affected; the AUSCAN score reflecting the three dimensions of pain, disability, and joint stiffness; and, to a lesser extent, the patient’s sex (with being female increasing the magnitude of the discomfort) and a duration of HOA >10 years. The association of erosive HOA with increased aesthetic discomfort is in line with Hodkinson et al.’s findings [11] and is in close agreement with several publications showing that the subset of patients who develop erosive HOA usually face a higher clinical burden, including a lower HRQoL, and have poorer radiographic outcomes [33–35] than patients without erosive HOA. It also seems logical that patients with the highest impact of pain, function, and stiffness reflecting more severe disease, as well as those with the most severe radiological damage corresponding to the presence of osteophytes, periarticular ossicles, sclerosis of subchondral bone, and pseudocystic areas with sclerotic walls in the subchondral bone [20], are those with the most important deformations and those who perceive a greater degree of aesthetic damage at the fingers. In their univariate analysis, as we found in our present study, Hodkinson et al. [10] suggested that radiological and clinical severity are also associated with aesthetic complaints. They found an association of aesthetic discomfort with depression and anxiety scores or poor HRQoL, parameters that we did not identify as being associated with our patients’ perception of aesthetic damage. Nevertheless, Hodkinson et al.’s multivariate analysis also identified the presence of erosions and patient global assessment of disease as the best predictors of high aesthetic concern. AUSCAN and K-L grading were not assessed in that particular study [11].

When we specifically compared patients with HAD with those with LAD, we identified the same parameters (i.e., radiological score, AUSCAN score, and presence of erosive OA) as being more prominent in patients with HAD. This finding is also in close agreement with the observations made by Hodkinson et al. [11].

Some weaknesses of our study have to be considered. First, we included a population of patients attending a bone and joint clinic. The vast majority of them did not have HOA as their main complaint. It should be kept in mind that, for some patients with HOA, symptoms may not be severe enough to seek consultation with a physician. In addition, individuals with HAD might also bypass a primary care physician, rheumatologist, and/or physical medicine and rehabilitation specialist and instead go directly to a hand surgeon. These patients are not covered by our analysis.

It should also be acknowledged that our population included a higher proportion of patients with erosive HOA (42.9 %) than reported in some epidemiological studies (5–15 %) [4, 6], even though this percentage is similar to the 45.9 % reported in the only other published study in which researchers investigated the assessment and determinants of aesthetic discomfort in HOA [11]. This might have to be considered when extrapolating our data to the general population with HOA.

It would also be of interest to consider the magnitude and determinants of aesthetic discomfort in a control group free of OA or in a control group likely to experience aesthetic discomfort, such as RA. Another interesting research question would be to assess how various levels of aesthetic discomfort can impact well-known consequences of the disease (e.g., HRQoL, depressive mood, sleep disturbances).

Eventually, a prospective follow-up study of our cohort could confirm the role of the determinants of aesthetic discomfort that we identified as predictors of the progression of aesthetic discomfort and damage. This might help to define a particular phenotype of patients with HOA who might benefit from specifically tailored management to avoid such consequences of the disease.

## Conclusions

Our study reemphasizes that aesthetic discomfort and aesthetic damage are underestimated and underrated complaints in patients presenting with HOA. In our study, the major determinants of the impact of these symptoms are female sex; duration of OA >10 years, but chiefly erosive HOA; severe radiological lesions; and the impact of the disease on pain, function, and stiffness as evaluated using the AUSCAN. Further studies are needed to develop a specific tool to better assess aesthetic discomfort in HOA with sufficient clinimetric properties, including sensitivity to change. This will stimulate the development of medications to treat the unmet medical need generated by the aesthetic discomfort caused by HOA.

## Additional files

Additional file 1: Table S1. Radiological severity and impact of hand osteoarthritis on functional disability, health-related quality of life, and psychological status. (DOC 38 kb) Additional file 2: Table S2. Characteristics of patients reporting high (HAD) or low (LAD) (aesthetic discomfort). (DOC 55 kb)

## Abbreviations

AUSCAN: Australian Canadian Osteoarthritis Hand Index
BMI: body mass index
EQ-5D: EuroQol
FIHOA: Functional Index for Hand Osteoarthritis
HAD: high aesthetic discomfort
HADS: Hospital Anxiety and Depression Scale
HOA: hand osteoarthritis
HRQoL: health-related quality of life
K-L: Kellgren–Lawrence
LAD: low aesthetic discomfort
LIHOC: LIège Hand Osteoarthritis Cohort
OA: osteoarthritis
Q1–Q3: interquartile range (first and third quartiles)
RA: rheumatoid arthritis
SF-12: 12-item Short-Form Health Survey
VAS: visual analogue scale
