# Supplementary material for: Aesthetic discomfort in hand osteoarthritis: results from the LIège Hand Osteoarthritis Cohort (LIHOC)
Source: Arthritis Res Ther. 2015 Nov 30;17:346. doi: 10.1186/s13075-015-0807-y (PMC4666038; doi:10.1186/s13075-015-0807-y)
Supplement: Additional file 1: Table S1. — Radiological severity and impact of hand osteoarthritis on functional disability, health-related quality of life, and psychological status. (DOC 38 kb) [file 13075_2015_807_MOESM1_ESM.doc]

Table S1: Radiologic severity and impact of hand osteoarthritis on functional disability, health-related quality of life and psychological status.

| Variables | Median | Q1-Q3 |
| --- | --- | --- |
| EQ-5D score (0-1) | 0.6 | 0.3-0.7 |
| SF-12 Physical score | 37.7 | 30.6-45.7 |
| SF-12 Mental score | 46.0 | 35.0-54.9 |
| AUSCAN Total score normalized (0-300) | 129.0 | 71.1-182.5 |
| FIHAO Total score (0-30) | 5.0 | 2.0-11.0 |
| HADS Anxiety (0-21) | 8.0 | 5.0-11.0 |
| HADS Depression (0-21) | 5.0 | 2.0-8.0 |
| RX Verbruggen and Veys Total score (0-218) | 31.6 | 20.9-48,2 |
| RX Kellgren-Lawrence Total score (0-128) | 54.0 | 40.0-66.0 |
