# Supplementary material for: Aesthetic discomfort in hand osteoarthritis: results from the LIège Hand Osteoarthritis Cohort (LIHOC)
Source: Arthritis Res Ther. 2015 Nov 30;17:346. doi: 10.1186/s13075-015-0807-y (PMC4666038; doi:10.1186/s13075-015-0807-y)
Supplement: Additional file 2: Table S2. — Characteristics of patients reporting high (HAD) or low (LAD) (aesthetic discomfort). (DOC 55 kb) [file 13075_2015_807_MOESM2_ESM.doc]

Table S2. Characteristics of patient reporting a high (HAD) or low (LAD) (aesthetic discomfort). p values refer to the outcomes of the univariate analysis comparing the two groups.

| **HAD (n=42)** | | | **LAD (n=100)** | | |
| --- | --- | --- | --- | --- | --- |
| **VARIABLES** | **Median** | **Q1-Q3** | **Median** | **Q1-Q3** | **p** |
| Age (Years) | 71,7 | 66,3-76,5 | 68,2 | 61,6-74,7 | 0,12 |
| BMI (Kg/m²) | 24,8 | 23,2-27,3 | 25,7 | 22,8-29,4 | 0,37 |
| Rx Verbruggen and Veys Total Score  (0-218) | 55,4 | 27,8-86,4 | 28,9 | 20,9-38-4 | <0,0001 |
| RX Kellgren-Lawrence  Total Score  (0-128) | 66,0 | 51,0-79,0 | 52,0 | 37,5-61,5 | <0,0001 |
| AUSCAN Total  Score Normalized  (0-300) | 160,6 | 118,7-227,8 | 106,6 | 53,6-169,5 | <0,0001 |
| FIHOA Total Score (0-30) | 9,5 | 4,0-15,0 | 4,0 | 2,0-9,0 | 0,0002 |
| Severe OA  (Number of Joints) | 10,0 | 7,0-16,0 | 3,5 | 0,0-8,0 | <0,0001 |
| Nodes (Number of Joints) | 11,0 | 8,0-16,0 | 8,5 | 5,0-12,5 | 0,012 |
| Swollen  (Number of Joints) | 3,0 | 1,0-4,0 | 2,0 | 1,0-4,0 | 0,34 |
| VAS Pain  (0-100) | 50,0 | 31,0-70,0 | 50,0 | 20,0-50,0 | 0,042 |
| Pain at rest  (Number of Joints) | 2,0 | 0,0-5,0 | 1,0 | 0,0-3,0 | 0,16 |
| Pain at pressure  (Number of Joints) | 6,5 | 3,0-15,0 | 3,0 | 1,0-7,0 | 0,0034 |
| Erosive OA  (Presence) | 73,8% |  | 27,0% |  | <0,0001 |
| Duration of OA  < 5 years  6-10 years  >10 years | 33,3%  16,7%  50,0% |  | 56,0%  22,0%  22,0% |  | 0,0038 |
| Gender  (Females) | 83,0% |  | 100% |  | 0,0044 |
